# Commentary: Epilepsy in Leigh Syndrome With Mitochondrial DNA Mutations

**DOI:** 10.3389/fneur.2019.00973

**Published:** 2019-10-21

**Authors:** Josef Finsterer

**Affiliations:** Krankenanstalt Rudolfstiftung, Messerli Institute, Vienna, Austria

**Keywords:** Leigh syndrome, epilepsy, mtDNA, oxidative phosphorylation, anti-seizure drugs, respiratory chain

With interest we read the article by Lee et al. about 25 patients with Leigh syndrome (LS) due to various mtDNA variants of whom 14 had epilepsy (1). We have the following comments and concerns.

A main shortcoming of the study is that heteroplasmy rates of the mtDNA variants were not provided. It is also not mentioned if mtDNA was extracted from blood lymphocytes, muscle, or from other tissues. Knowing heteroplasmy rates in various tissues is crucial as it may be one of the factors determining the phenotype and outcome.

LS may not only be due to mtDNA variants but also due to nDNA variants. We should be informed about which nDNA variants were found in the 100 patients who did not carry an mtDNA variant.

A shortcoming of the study is the absence of a comprehensive family history. We should know how many mothers of the 25 included patients were clinically affected or carried the mtDNA variant of their son. We should also know if there were other first degree relatives with epilepsy or phenotypic features of a mitochondrial disorder (MID). According to Table 1 the family history was negative for epilepsy in all 25 patients. This is quite unusual in the light that 75% of the MIDs due to mtDNA variants are maternally inherited (2).

In the abstract 14 of 25 patients had epilepsy but in the results section and Table 1 only 32% of the 25 patients had seizures (1). The authors should explain this discrepancy.

We should be informed why only 12 of the 14 patients with epilepsy received a treatment with anti-seizure drugs (ASDs). We should also know if the two patients without ASDs were seizure-free or if they or their parents refused ASD therapy.

We should know if the two patients on a ketogenic diet (KD) were the ones who did not receive ASD treatment. It should be mentioned if the KD was effective and reduced seizure frequency in the two patients on KD as has been previously reported (3).

Rarely, patients with LS may develop stroke-like episodes (SLEs) (4) of which the stroke-like lesion (SLL) is the morphological equivalent on imaging (5). We should know in how many of the 14 patients with epilepsy, epilepsy or seizures were associated with a SLE, since seizures may trigger SLEs and vice versa.

Interestingly, the authors mention that >50% of the 125 patients received ophthalmologic treatment (1). It should be indicted if patients had pigmentary retinopathy, glaucoma, cataract, optic atrophy, refractory errors, megalocornea, iris atrophy, pupillary dysfunction, cataract, ciliary body epithelial dysfunction, macular degeneration, retinal hypertrophy, choroidal atrophy, or corneal ulcers and conjunctivitis, frequently found in MID patients (6).

LS is frequently a disease with poor prognosis (7). Since mean follow-up duration was 5.7 years it is quite likely that some of the initially included 25 patients had deceased during follow-up. Thus, we should be informed how many of the 25 died during follow-up and which were the causes of death. According to Table 1, one third of the patients had cardiac and one third had pulmonary involvement, which may significantly contribute to mortality of these patients.

Overall, the study could be more meaningful if the various shortcomings were solved and if more data about family history, multiorgan involvement, mutation load, and outcome were provided.

## Author Contributions

JF contributed to the design, literature search, discussion, first draft, and critical comments.

### Conflict of Interest Statement

The author declares that the research was conducted in the absence of any commercial or financial relationships that could be construed as a potential conflict of interest.
